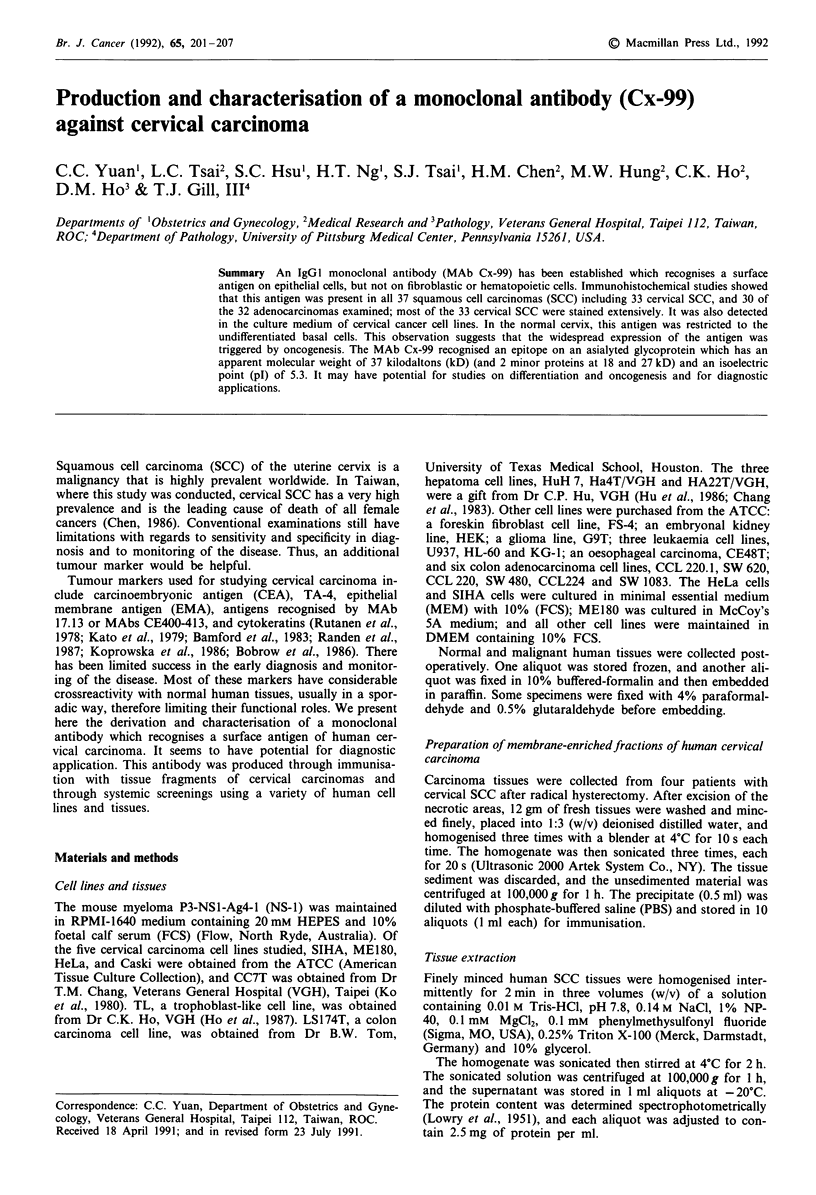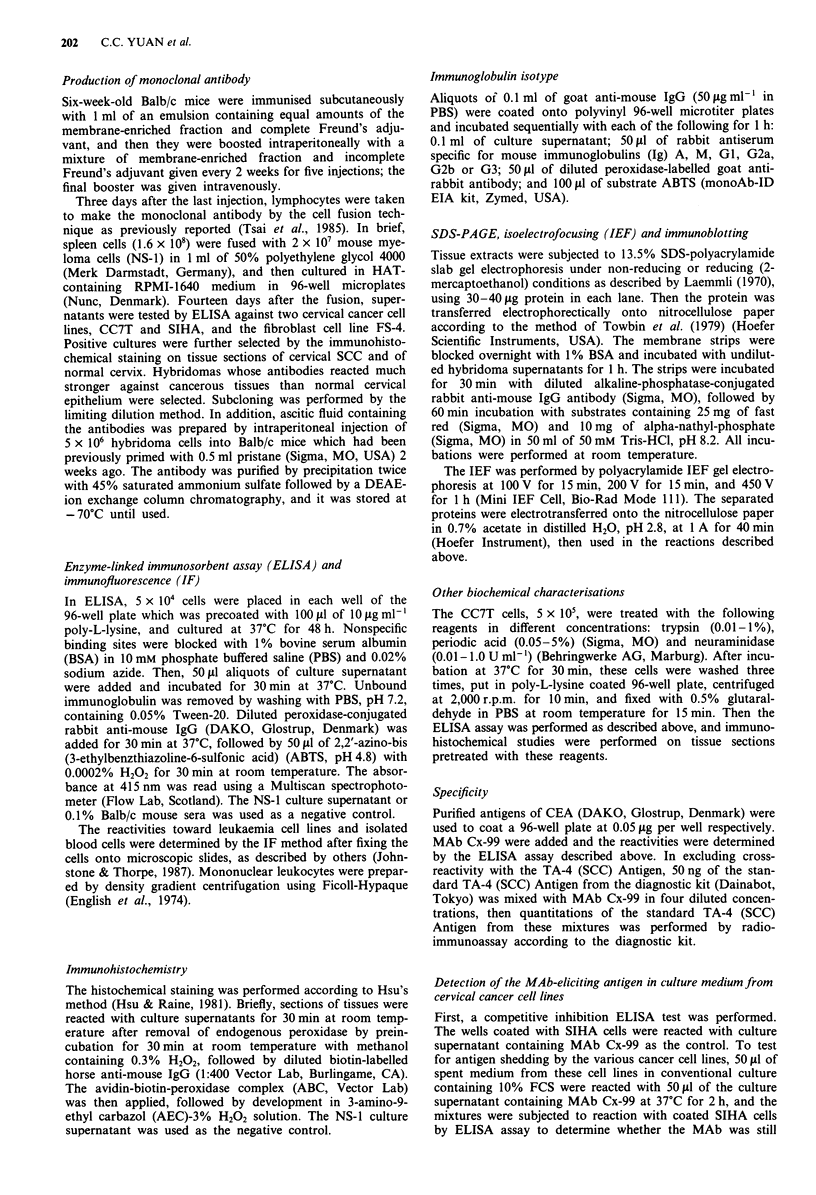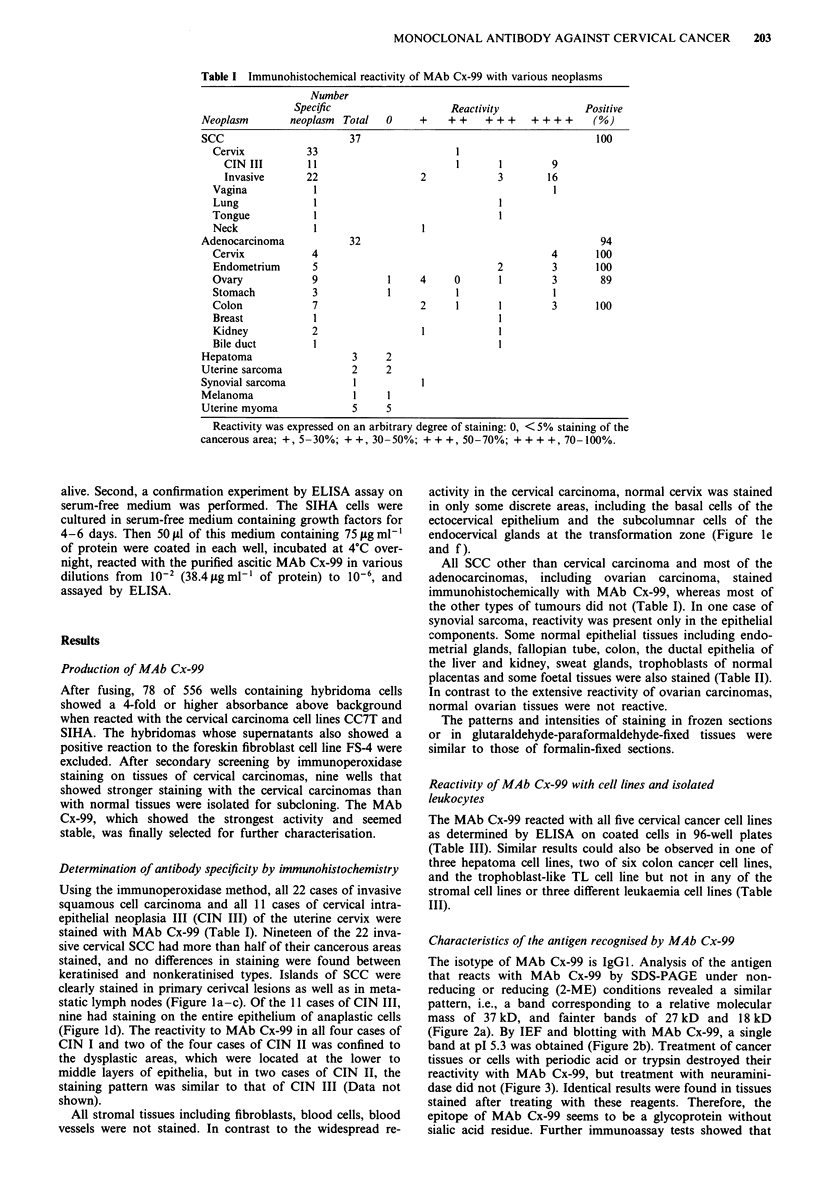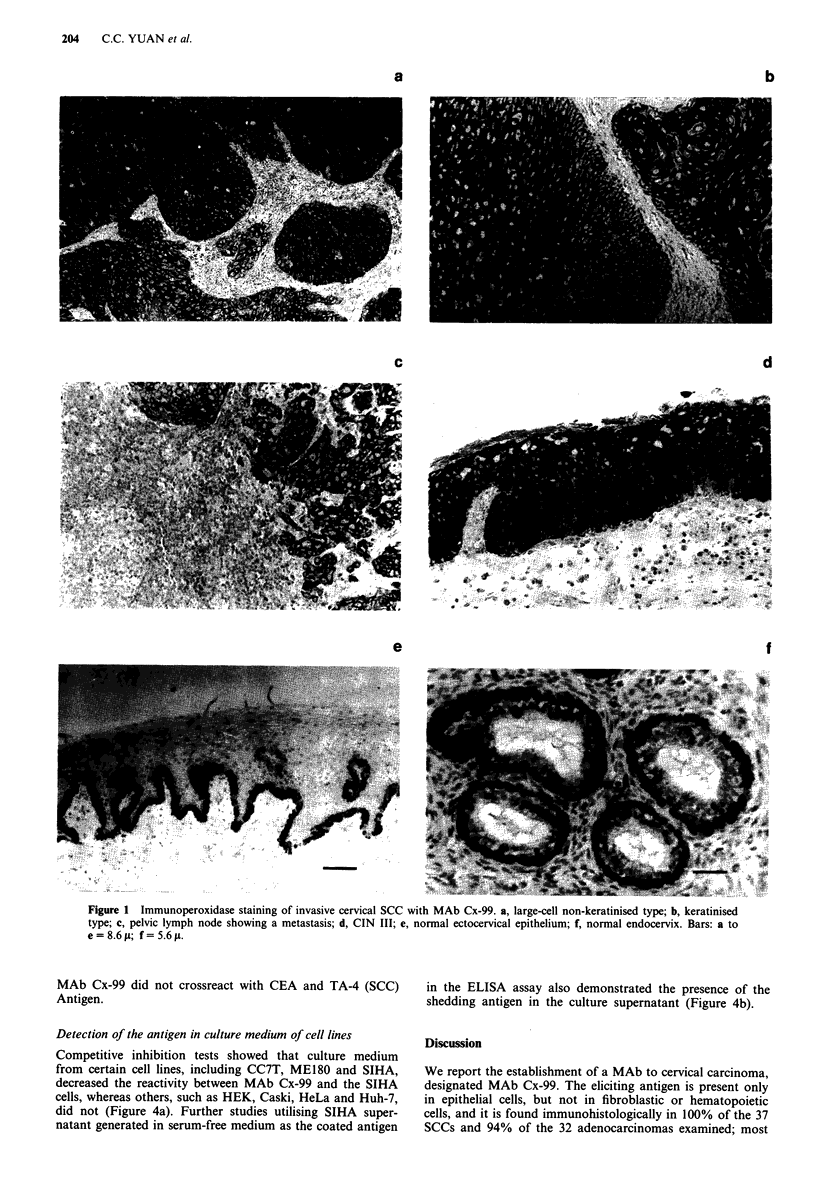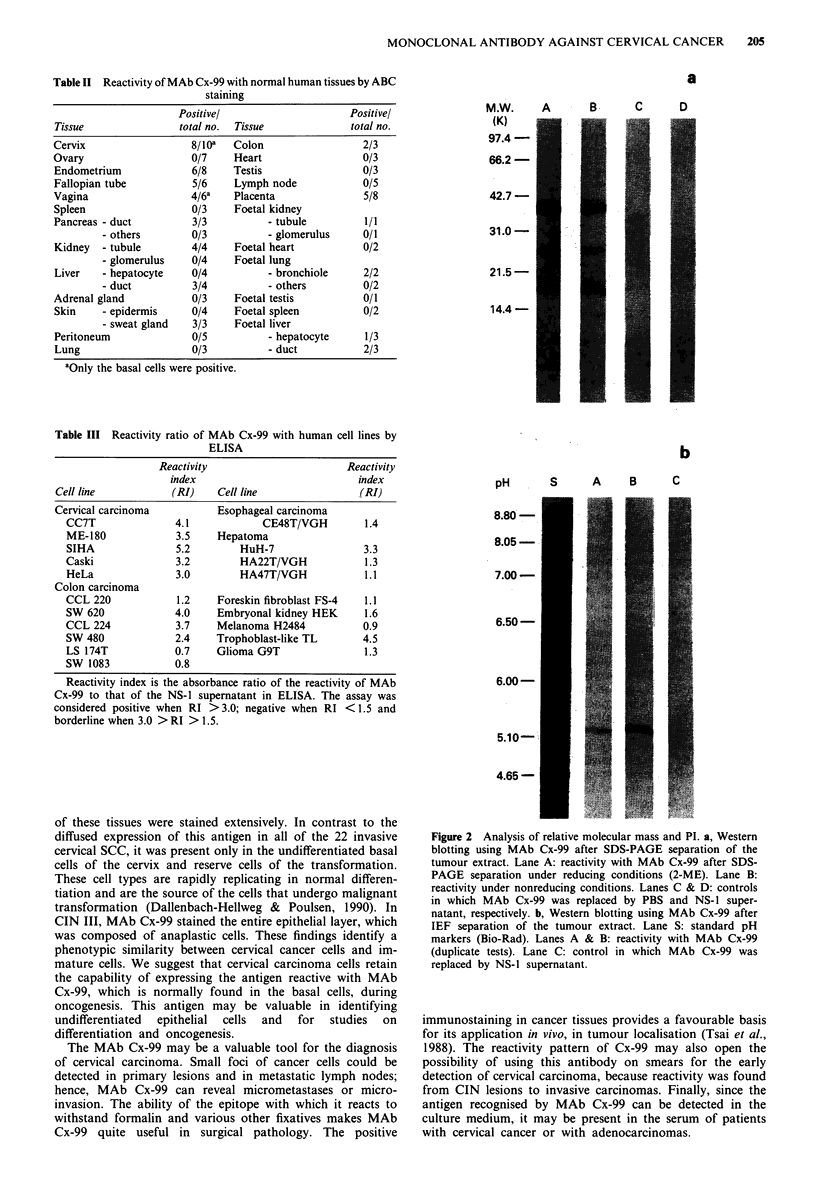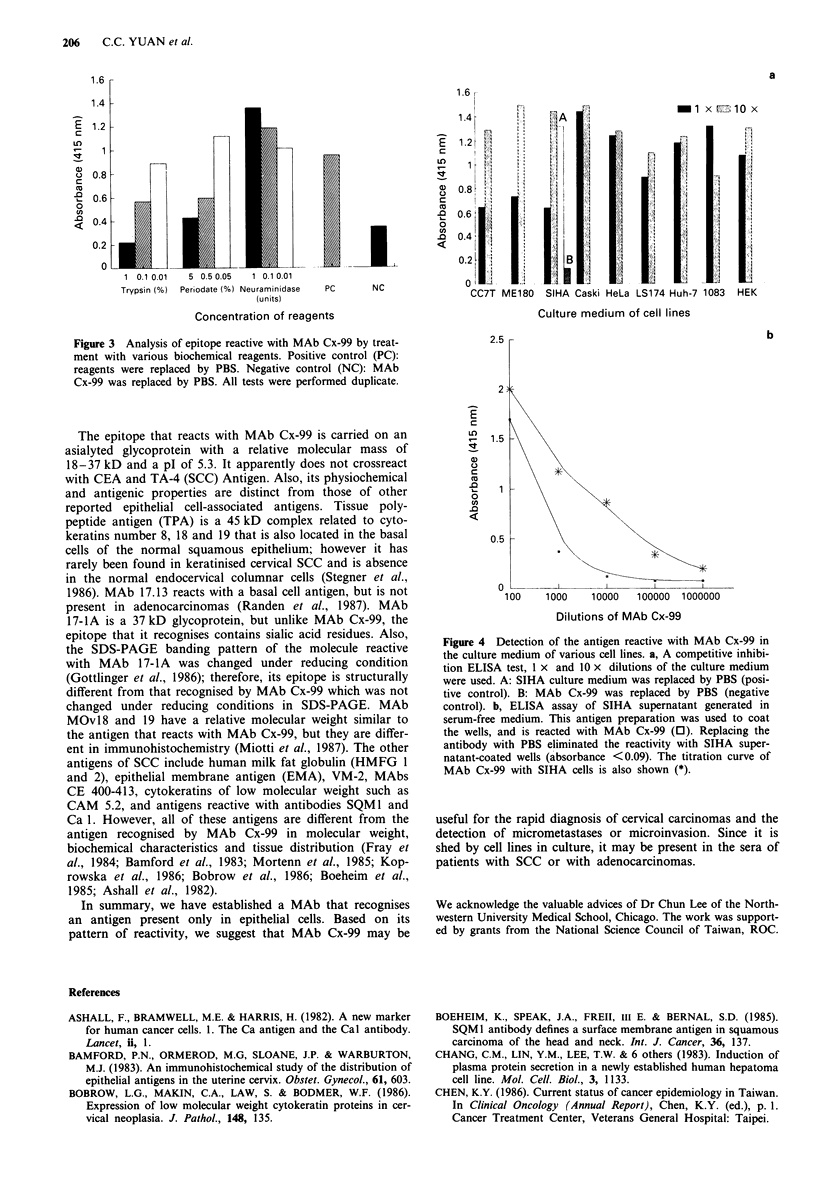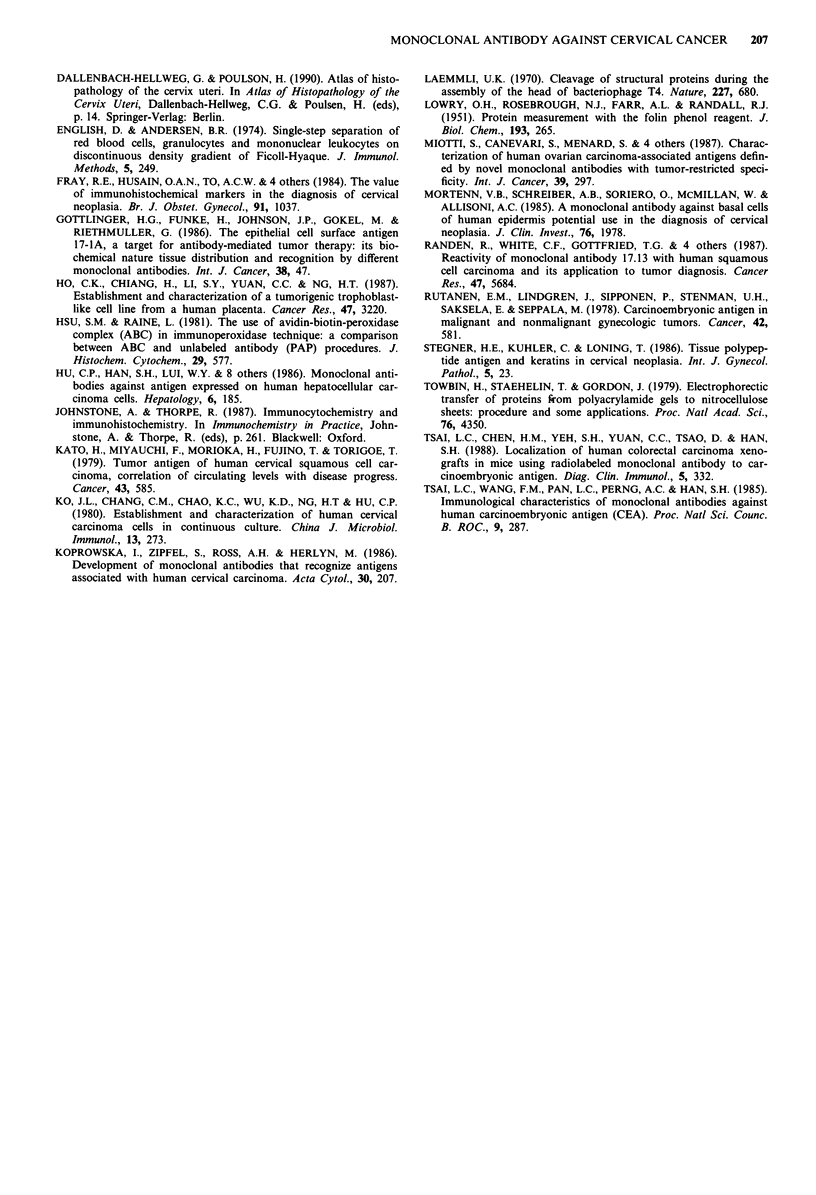# Production and characterisation of a monoclonal antibody (Cx-99) against cervical carcinoma.

**DOI:** 10.1038/bjc.1992.42

**Published:** 1992-02

**Authors:** C. C. Yuan, L. C. Tsai, S. C. Hsu, H. T. Ng, S. J. Tsai, H. M. Chen, M. W. Hung, C. K. Ho, D. M. Ho, T. J. Gill

**Affiliations:** Department of Obstetrics and Gynecology, Veterans General Hospital, Taipei, Taiwan, ROC.

## Abstract

**Images:**


Br. J. Cancer (1992), 65, 201-207 © Macmillan Press Ltd., 1992

### Production and characterisation of a monoclonal antibody (Cx-99) against cervical carcinoma

C.C. Yuan[1], L.C. Tsai[2], S.C. Hsu[1], H.T. Ng[1], S.J. Tsai[1], H.M. Chen[2], M.W. Hung[2], C.K. Ho[2], D.M. Ho[3] & T.J. Gill, III[4]

*Departments of [1]Obstetrics and Gynecology, [2]Medical Research and [3]Pathology, Veterans General Hospital, Taipei 112, Taiwan, ROC; [4]Department of Pathology, University of Pittsburg Medical Center, Pennsylvania 15261, USA.*

**Summary** An IgG1 monoclonal antibody (MAb Cx-99) has been established which recognises a surface antigen on epithelial cells, but not on fibroblastic or hematopoietic cells. Immunohistochemical studies showed that this antigen was present in all 37 squamous cell carcinomas (SCC) including 33 cervical SCC, and 30 of the 32 adenocarcinomas examined; most of the 33 cervical SCC were stained extensively. It was also detected in the culture medium of cervical cancer cell lines. In the normal cervix, this antigen was restricted to the undifferentiated basal cells. This observation suggests that the widespread expression of the antigen was triggered by oncogenesis. The MAb Cx-99 recognised an epitope on an asialyted glycoprotein which has an apparent molecular weight of 37 kilodaltons (kD) (and 2 minor proteins at 18 and 27 kD) and an isoelectric point (pI) of 5.3. It may have potential for studies on differentiation and oncogenesis and for diagnostic applications.

Squamous cell carcinoma (SCC) of the uterine cervix is a malignancy that is highly prevalent worldwide. In Taiwan, where this study was conducted, cervical SCC has a very high prevalence and is the leading cause of death of all female cancers (Chen, 1986). Conventional examinations still have limitations with regards to sensitivity and specificity in diagnosis and to monitoring of the disease. Thus, an additional tumour marker would be helpful.

Tumour markers used for studying cervical carcinoma include carcinoembryonic antigen (CEA), TA-4, epithelial membrane antigen (EMA), antigens recognised by MAb 17.13 or MAbs CE400-413, and cytokeratins (Rutanen *et al.*, 1978; Kato *et al.*, 1979; Bamford *et al.*, 1983; Randen *et al.*, 1987; Koprowska *et al.*, 1986; Bobrow *et al.*, 1986). There has been limited success in the early diagnosis and monitoring of the disease. Most of these markers have considerable crossreactivity with normal human tissues, usually in a sporadic way, therefore limiting their functional roles. We present here the derivation and characterisation of a monoclonal antibody which recognises a surface antigen of human cervical carcinoma. It seems to have potential for diagnostic application. This antibody was produced through immunisation with tissue fragments of cervical carcinomas and through systemic screenings using a variety of human cell lines and tissues.

### Materials and methods

### *Cell lines and tissues*

The mouse myeloma P3-NS1-Ag4-1 (NS-1) was maintained in RPMI-1640 medium containing 20 mM HEPES and 10% foetal calf serum (FCS) (Flow, North Ryde, Australia). Of the five cervical carcinoma cell lines studied, SIHA, ME180, HeLa, and Caski were obtained from the ATCC (American Tissue Culture Collection), and CC7T was obtained from Dr T.M. Chang, Veterans General Hospital (VGH), Taipei (Ko *et al.*, 1980). TL, a trophoblast-like cell line, was obtained from Dr C.K. Ho, VGH (Ho *et al.*, 1987). LS174T, a colon carcinoma cell line, was obtained from Dr B.W. Tom, University of Texas Medical School, Houston. The three hepatoma cell lines, HuH 7, Ha4T/VGH and HA22T/VGH, were a gift from Dr C.P. Hu, VGH (Hu *et al.*, 1986; Chang *et al.*, 1983). Other cell lines were purchased from the ATCC: a foreskin fibroblast cell line, FS-4; an embryonal kidney line, HEK; a glioma line, G9T; three leukaemia cell lines, U937, HL-60 and KG-1; an oesophageal carcinoma, CE48T; and six colon adenocarcinoma cell lines, CCL 220.1, SW 620, CCL 220, SW 480, CCL224 and SW 1083. The HeLa cells and SIHA cells were cultured in minimal essential medium (MEM) with 10% (FCS); ME180 was cultured in McCoy's 5A medium; and all other cell lines were maintained in DMEM containing 10% FCS.

Normal and malignant human tissues were collected post-operatively. One aliquot was stored frozen, and another aliquot was fixed in 10% buffered-formalin and then embedded in paraffin. Some specimens were fixed with 4% paraformaldehyde and 0.5% glutaraldehyde before embedding.

### *Preparation of membrane-enriched fractions of human cervical carcinoma*

Carcinoma tissues were collected from four patients with cervical SCC after radical hysterectomy. After excision of the necrotic areas, 12 gm of fresh tissues were washed and minced finely, placed into 1:3 (w/v) deionised distilled water, and homogenised three times with a blender at 4°C for 10 s each time. The homogenate was then sonicated three times, each for 20 s (Ultrasonic 2000 Artek System Co., NY). The tissue sediment was discarded, and the unsedimented material was centrifuged at 100,000 *g* for 1 h. The precipitate (0.5 ml) was diluted with phosphate-buffered saline (PBS) and stored in 10 aliquots (1 ml each) for immunisation.

### *Tissue extraction*

Finely minced human SCC tissues were homogenised intermittently for 2 min in three volumes (w/v) of a solution containing 0.01 M Tris-HCl, pH 7.8, 0.14 M NaCl, 1% NP-40, 0.1 mM $MgCl_2$, 0.1 mM phenylmethysulfonyl fluoride (Sigma, MO, USA), 0.25% Triton X-100 (Merck, Darmstadt, Germany) and 10% glycerol.

The homogenate was sonicated then stirred at 4°C for 2 h. The sonicated solution was centrifuged at 100,000 *g* for 1 h, and the supernatant was stored in 1 ml aliquots at −20°C. The protein content was determined spectrophotometrically (Lowry *et al.*, 1951), and each aliquot was adjusted to contain 2.5 mg of protein per ml.

Correspondence: C.C. Yuan, Department of Obstetrics and Gynecology, Veterans General Hospital, Taipei 112, Taiwan, ROC.
Received 18 April 1991; and in revised form 23 July 1991.

*Production of monoclonal antibody*

Six-week-old Balb/c mice were immunised subcutaneously with 1 ml of an emulsion containing equal amounts of the membrane-enriched fraction and complete Freund's adjuvant, and then they were boosted intraperitoneally with a mixture of membrane-enriched fraction and incomplete Freund's adjuvant given every 2 weeks for five injections; the final booster was given intravenously.

Three days after the last injection, lymphocytes were taken to make the monoclonal antibody by the cell fusion technique as previously reported (Tsai *et al.*, 1985). In brief, spleen cells ($1.6 \times 10^8$) were fused with $2 \times 10^7$ mouse myeloma cells (NS-1) in 1 ml of 50% polyethylene glycol 4000 (Merk Darmstadt, Germany), and then cultured in HAT-containing RPMI-1640 medium in 96-well microplates (Nunc, Denmark). Fourteen days after the fusion, supernatants were tested by ELISA against two cervical cancer cell lines, CC7T and SIHA, and the fibroblast cell line FS-4. Positive cultures were further selected by the immunohistochemical staining on tissue sections of cervical SCC and of normal cervix. Hybridomas whose antibodies reacted much stronger against cancerous tissues than normal cervical epithelium were selected. Subcloning was performed by the limiting dilution method. In addition, ascitic fluid containing the antibodies was prepared by intraperitoneal injection of $5 \times 10^6$ hybridoma cells into Balb/c mice which had been previously primed with 0.5 ml pristane (Sigma, MO, USA) 2 weeks ago. The antibody was purified by precipitation twice with 45% saturated ammonium sulfate followed by a DEAE-ion exchange column chromatography, and it was stored at $-70°C$ until used.

*Enzyme-linked immunosorbent assay (ELISA) and immunofluorescence (IF)*

In ELISA, $5 \times 10^4$ cells were placed in each well of the 96-well plate which was precoated with 100 µl of 10 µg ml$^{-1}$ poly-L-lysine, and cultured at 37°C for 48 h. Nonspecific binding sites were blocked with 1% bovine serum albumin (BSA) in 10 mM phosphate buffered saline (PBS) and 0.02% sodium azide. Then, 50 µl aliquots of culture supernatant were added and incubated for 30 min at 37°C. Unbound immunoglobulin was removed by washing with PBS, pH 7.2, containing 0.05% Tween-20. Diluted peroxidase-conjugated rabbit anti-mouse IgG (DAKO, Glostrup, Denmark) was added for 30 min at 37°C, followed by 50 µl of 2,2'-azino-bis (3-ethylbenzthiazoline-6-sulfonic acid) (ABTS, pH 4.8) with 0.0002% $H_2O_2$ for 30 min at room temperature. The absorbance at 415 nm was read using a Multiscan spectrophotometer (Flow Lab, Scotland). The NS-1 culture supernatant or 0.1% Balb/c mouse sera was used as a negative control.

The reactivities toward leukaemia cell lines and isolated blood cells were determined by the IF method after fixing the cells onto microscopic slides, as described by others (Johnstone & Thorpe, 1987). Mononuclear leukocytes were prepared by density gradient centrifugation using Ficoll-Hypaque (English *et al.*, 1974).

*Immunohistochemistry*

The histochemical staining was performed according to Hsu's method (Hsu & Raine, 1981). Briefly, sections of tissues were reacted with culture supernatants for 30 min at room temperature after removal of endogenous peroxidase by preincubation for 30 min at room temperature with methanol containing 0.3% $H_2O_2$, followed by diluted biotin-labelled horse anti-mouse IgG (1:400 Vector Lab, Burlingame, CA). The avidin-biotin-peroxidase complex (ABC, Vector Lab) was then applied, followed by development in 3-amino-9-ethyl carbazol (AEC)-3% $H_2O_2$ solution. The NS-1 culture supernatant was used as the negative control.

*Immunoglobulin isotype*

Aliquots of 0.1 ml of goat anti-mouse IgG (50 µg ml$^{-1}$ in PBS) were coated onto polyvinyl 96-well microtiter plates and incubated sequentially with each of the following for 1 h: 0.1 ml of culture supernatant; 50 µl of rabbit antiserum specific for mouse immunoglobulins (Ig) A, M, G1, G2a, G2b or G3; 50 µl of diluted peroxidase-labelled goat anti-rabbit antibody; and 100 µl of substrate ABTS (monoAb-ID EIA kit, Zymed, USA).

*SDS-PAGE, isoelectrofocusing (IEF) and immunoblotting*

Tissue extracts were subjected to 13.5% SDS-polyacrylamide slab gel electrophoresis under non-reducing or reducing (2-mercaptoethanol) conditions as described by Laemmli (1970), using 30–40 µg protein in each lane. Then the protein was transferred electrophorectically onto nitrocellulose paper according to the method of Towbin *et al.* (1979) (Hoefer Scientific Instruments, USA). The membrane strips were blocked overnight with 1% BSA and incubated with undiluted hybridoma supernatants for 1 h. The strips were incubated for 30 min with diluted alkaline-phosphatase-conjugated rabbit anti-mouse IgG antibody (Sigma, MO), followed by 60 min incubation with substrates containing 25 mg of fast red (Sigma, MO) and 10 mg of alpha-nathyl-phosphate (Sigma, MO) in 50 ml of 50 mM Tris-HCl, pH 8.2. All incubations were performed at room temperature.

The IEF was performed by polyacrylamide IEF gel electrophoresis at 100 V for 15 min, 200 V for 15 min, and 450 V for 1 h (Mini IEF Cell, Bio-Rad Mode 111). The separated proteins were electrotransferred onto the nitrocellulose paper in 0.7% acetate in distilled $H_2O$, pH 2.8, at 1 A for 40 min (Hoefer Instrument), then used in the reactions described above.

*Other biochemical characterisations*

The CC7T cells, $5 \times 10^5$, were treated with the following reagents in different concentrations: trypsin (0.01–1%), periodic acid (0.05–5%) (Sigma, MO) and neuraminidase (0.01–1.0 U ml$^{-1}$) (Behringwerke AG, Marburg). After incubation at 37°C for 30 min, these cells were washed three times, put in poly-L-lysine coated 96-well plate, centrifuged at 2,000 r.p.m. for 10 min, and fixed with 0.5% glutaraldehyde in PBS at room temperature for 15 min. Then the ELISA assay was performed as described above, and immunohistochemical studies were performed on tissue sections pretreated with these reagents.

*Specificity*

Purified antigens of CEA (DAKO, Glostrup, Denmark) were used to coat a 96-well plate at 0.05 µg per well respectively. MAb Cx-99 were added and the reactivities were determined by the ELISA assay described above. In excluding cross-reactivity with the TA-4 (SCC) Antigen, 50 ng of the standard TA-4 (SCC) Antigen from the diagnostic kit (Dainabot, Tokyo) was mixed with MAb Cx-99 in four diluted concentrations, then quantitations of the standard TA-4 (SCC) Antigen from these mixtures was performed by radioimmunoassay according to the diagnostic kit.

*Detection of the MAb-eliciting antigen in culture medium from cervical cancer cell lines*

First, a competitive inhibition ELISA test was performed. The wells coated with SIHA cells were reacted with culture supernatant containing MAb Cx-99 as the control. To test for antigen shedding by the various cancer cell lines, 50 µl of spent medium from these cell lines in conventional culture containing 10% FCS were reacted with 50 µl of the culture supernatant containing MAb Cx-99 at 37°C for 2 h, and the mixtures were subjected to reaction with coated SIHA cells by ELISA assay to determine whether the MAb was still

**Table I** Immunohistochemical reactivity of MAb Cx-99 with various neoplasms

| *Neoplasm* | *Number Specific neoplasm* | *Total* | *Reactivity* 0 | + | ++ | +++ | ++++ | *Positive (%)* |
|---|---|---|---|---|---|---|---|---|
| SCC | | 37 | | | | | | 100 |
| Cervix | 33 | | | | 1 | | | |
| CIN III | 11 | | | | 1 | 1 | 9 | |
| Invasive | 22 | | | 2 | | 3 | 16 | |
| Vagina | 1 | | | | | | 1 | |
| Lung | 1 | | | | | 1 | | |
| Tongue | 1 | | | | | 1 | | |
| Neck | 1 | | | 1 | | | | |
| Adenocarcinoma | | 32 | | | | | | 94 |
| Cervix | 4 | | | | | | 4 | 100 |
| Endometrium | 5 | | | | | 2 | 3 | 100 |
| Ovary | 9 | | 1 | 4 | 0 | 1 | 3 | 89 |
| Stomach | 3 | | 1 | | 1 | | 1 | |
| Colon | 7 | | | 2 | 1 | 1 | 3 | 100 |
| Breast | 1 | | | | | 1 | | |
| Kidney | 2 | | | 1 | | 1 | | |
| Bile duct | 1 | | | | | 1 | | |
| Hepatoma | | 3 | 2 | | | | | |
| Uterine sarcoma | | 2 | 2 | | | | | |
| Synovial sarcoma | | 1 | | 1 | | | | |
| Melanoma | | 1 | 1 | | | | | |
| Uterine myoma | | 5 | 5 | | | | | |

Reactivity was expressed on an arbitrary degree of staining: 0, <5% staining of the cancerous area; +, 5–30%; ++, 30–50%; +++, 50–70%; ++++, 70–100%.

alive. Second, a confirmation experiment by ELISA assay on serum-free medium was performed. The SIHA cells were cultured in serum-free medium containing growth factors for 4–6 days. Then 50 µl of this medium containing 75 µg ml$^{-1}$ of protein were coated in each well, incubated at 4°C overnight, reacted with the purified ascitic MAb Cx-99 in various dilutions from $10^{-2}$ (38.4 µg ml$^{-1}$ of protein) to $10^{-6}$, and assayed by ELISA.

### Results

### *Production of MAb Cx-99*

After fusing, 78 of 556 wells containing hybridoma cells showed a 4-fold or higher absorbance above background when reacted with the cervical carcinoma cell lines CC7T and SIHA. The hybridomas whose supernatants also showed a positive reaction to the foreskin fibroblast cell line FS-4 were excluded. After secondary screening by immunoperoxidase staining on tissues of cervical carcinomas, nine wells that showed stronger staining with the cervical carcinomas than with normal tissues were isolated for subcloning. The MAb Cx-99, which showed the strongest activity and seemed stable, was finally selected for further characterisation.

### *Determination of antibody specificity by immunohistochemistry*

Using the immunoperoxidase method, all 22 cases of invasive squamous cell carcinoma and all 11 cases of cervical intraepithelial neoplasia III (CIN III) of the uterine cervix were stained with MAb Cx-99 (Table I). Nineteen of the 22 invasive cervical SCC had more than half of their cancerous areas stained, and no differences in staining were found between keratinised and nonkeratinised types. Islands of SCC were clearly stained in primary cerivcal lesions as well as in metastatic lymph nodes (Figure 1a–c). Of the 11 cases of CIN III, nine had staining on the entire epithelium of anaplastic cells (Figure 1d). The reactivity to MAb Cx-99 in all four cases of CIN I and two of the four cases of CIN II was confined to the dysplastic areas, which were located at the lower to middle layers of epithelia, but in two cases of CIN II, the staining pattern was similar to that of CIN III (Data not shown).

All stromal tissues including fibroblasts, blood cells, blood vessels were not stained. In contrast to the widespread reactivity in the cervical carcinoma, normal cervix was stained in only some discrete areas, including the basal cells of the ectocervical epithelium and the subcolumnar cells of the endocervical glands at the transformation zone (Figure 1e and f).

All SCC other than cervical carcinoma and most of the adenocarcinomas, including ovarian carcinoma, stained immunohistochemically with MAb Cx-99, whereas most of the other types of tumours did not (Table I). In one case of synovial sarcoma, reactivity was present only in the epithelial components. Some normal epithelial tissues including endometrial glands, fallopian tube, colon, the ductal epithelia of the liver and kidney, sweat glands, trophoblasts of normal placentas and some foetal tissues were also stained (Table II). In contrast to the extensive reactivity of ovarian carcinomas, normal ovarian tissues were not reactive.

The patterns and intensities of staining in frozen sections or in glutaraldehyde-paraformaldehyde-fixed tissues were similar to those of formalin-fixed sections.

### *Reactivity of MAb Cx-99 with cell lines and isolated leukocytes*

The MAb Cx-99 reacted with all five cervical cancer cell lines as determined by ELISA on coated cells in 96-well plates (Table III). Similar results could also be observed in one of three hepatoma cell lines, two of six colon cancer cell lines, and the trophoblast-like TL cell line but not in any of the stromal cell lines or three different leukaemia cell lines (Table III).

### *Characteristics of the antigen recognised by MAb Cx-99*

The isotype of MAb Cx-99 is IgG1. Analysis of the antigen that reacts with MAb Cx-99 by SDS-PAGE under non-reducing or reducing (2-ME) conditions revealed a similar pattern, i.e., a band corresponding to a relative molecular mass of 37 kD, and fainter bands of 27 kD and 18 kD (Figure 2a). By IEF and blotting with MAb Cx-99, a single band at pI 5.3 was obtained (Figure 2b). Treatment of cancer tissues or cells with periodic acid or trypsin destroyed their reactivity with MAb Cx-99, but treatment with neuraminidase did not (Figure 3). Identical results were found in tissues stained after treating with these reagents. Therefore, the epitope of MAb Cx-99 seems to be a glycoprotein without sialic acid residue. Further immunoassay tests showed that

**Figure 1** Immunoperoxidase staining of invasive cervical SCC with MAb Cx-99. **a**, large-cell non-keratinised type; **b**, keratinised type; **c**, pelvic lymph node showing a metastasis; **d**, CIN III; **e**, normal ectocervical epithelium; **f**, normal endocervix. Bars: **a** to **e** = 8.6 μ; **f** = 5.6 μ.

MAb Cx-99 did not crossreact with CEA and TA-4 (SCC) Antigen.

*Detection of the antigen in culture medium of cell lines*

Competitive inhibition tests showed that culture medium from certain cell lines, including CC7T, ME180 and SIHA, decreased the reactivity between MAb Cx-99 and the SIHA cells, whereas others, such as HEK, Caski, HeLa and Huh-7, did not (Figure 4a). Further studies utilising SIHA supernatant generated in serum-free medium as the coated antigen in the ELISA assay also demonstrated the presence of the shedding antigen in the culture supernatant (Figure 4b).

### Discussion

We report the establishment of a MAb to cervical carcinoma, designated MAb Cx-99. The eliciting antigen is present only in epithelial cells, but not in fibroblastic or hematopoietic cells, and it is found immunohistologically in 100% of the 37 SCCs and 94% of the 32 adenocarcinomas examined; most

**Table II** Reactivity of MAb Cx-99 with normal human tissues by ABC staining

| *Tissue* | *Positive/ total no.* | *Tissue* | *Positive/ total no.* |
|---|---|---|---|
| Cervix | 8/10[a] | Colon | 2/3 |
| Ovary | 0/7 | Heart | 0/3 |
| Endometrium | 6/8 | Testis | 0/3 |
| Fallopian tube | 5/6 | Lymph node | 0/5 |
| Vagina | 4/6[a] | Placenta | 5/8 |
| Spleen | 0/3 | Foetal kidney | |
| Pancreas - duct | 3/3 | - tubule | 1/1 |
| - others | 0/3 | - glomerulus | 0/1 |
| Kidney - tubule | 4/4 | Foetal heart | 0/2 |
| - glomerulus | 0/4 | Foetal lung | |
| Liver - hepatocyte | 0/4 | - bronchiole | 2/2 |
| - duct | 3/4 | - others | 0/2 |
| Adrenal gland | 0/3 | Foetal testis | 0/1 |
| Skin - epidermis | 0/4 | Foetal spleen | 0/2 |
| - sweat gland | 3/3 | Foetal liver | |
| Peritoneum | 0/5 | - hepatocyte | 1/3 |
| Lung | 0/3 | - duct | 2/3 |

[a]Only the basal cells were positive.

**Table III** Reactivity ratio of MAb Cx-99 with human cell lines by ELISA

| *Cell line* | *Reactivity index (RI)* | *Cell line* | *Reactivity index (RI)* |
|---|---|---|---|
| Cervical carcinoma | | Esophageal carcinoma | |
| CC7T | 4.1 | CE48T/VGH | 1.4 |
| ME-180 | 3.5 | Hepatoma | |
| SIHA | 5.2 | HuH-7 | 3.3 |
| Caski | 3.2 | HA22T/VGH | 1.3 |
| HeLa | 3.0 | HA47T/VGH | 1.1 |
| Colon carcinoma | | | |
| CCL 220 | 1.2 | Foreskin fibroblast FS-4 | 1.1 |
| SW 620 | 4.0 | Embryonal kidney HEK | 1.6 |
| CCL 224 | 3.7 | Melanoma H2484 | 0.9 |
| SW 480 | 2.4 | Trophoblast-like TL | 4.5 |
| LS 174T | 0.7 | Glioma G9T | 1.3 |
| SW 1083 | 0.8 | | |

Reactivity index is the absorbance ratio of the reactivity of MAb Cx-99 to that of the NS-1 supernatant in ELISA. The assay was considered positive when RI $>3.0$; negative when RI $<1.5$ and borderline when $3.0 > \text{RI} > 1.5$.

of these tissues were stained extensively. In contrast to the diffused expression of this antigen in all of the 22 invasive cervical SCC, it was present only in the undifferentiated basal cells of the cervix and reserve cells of the transformation. These cell types are rapidly replicating in normal differentiation and are the source of the cells that undergo malignant transformation (Dallenbach-Hellweg & Poulsen, 1990). In CIN III, MAb Cx-99 stained the entire epithelial layer, which was composed of anaplastic cells. These findings identify a phenotypic similarity between cervical cancer cells and immature cells. We suggest that cervical carcinoma cells retain the capability of expressing the antigen reactive with MAb Cx-99, which is normally found in the basal cells, during oncogenesis. This antigen may be valuable in identifying undifferentiated epithelial cells and for studies on differentiation and oncogenesis.

The MAb Cx-99 may be a valuable tool for the diagnosis of cervical carcinoma. Small foci of cancer cells could be detected in primary lesions and in metastatic lymph nodes; hence, MAb Cx-99 can reveal micrometastases or microinvasion. The ability of the epitope with which it reacts to withstand formalin and various other fixatives makes MAb Cx-99 quite useful in surgical pathology. The positive immunostaining in cancer tissues provides a favourable basis for its application *in vivo*, in tumour localisation (Tsai *et al.*, 1988). The reactivity pattern of Cx-99 may also open the possibility of using this antibody on smears for the early detection of cervical carcinoma, because reactivity was found from CIN lesions to invasive carcinomas. Finally, since the antigen recognised by MAb Cx-99 can be detected in the culture medium, it may be present in the serum of patients with cervical cancer or with adenocarcinomas.

**Figure 2** Analysis of relative molecular mass and PI. **a**, Western blotting using MAb Cx-99 after SDS-PAGE separation of the tumour extract. Lane A: reactivity with MAb Cx-99 after SDS-PAGE separation under reducing conditions (2-ME). Lane B: reactivity under nonreducing conditions. Lanes C & D: controls in which MAb Cx-99 was replaced by PBS and NS-1 supernatant, respectively. **b**, Western blotting using MAb Cx-99 after IEF separation of the tumour extract. Lane S: standard pH markers (Bio-Rad). Lanes A & B: reactivity with MAb Cx-99 (duplicate tests). Lane C: control in which MAb Cx-99 was replaced by NS-1 supernatant.

**Figure 3** Analysis of epitope reactive with MAb Cx-99 by treatment with various biochemical reagents. Positive control (PC): reagents were replaced by PBS. Negative control (NC): MAb Cx-99 was replaced by PBS. All tests were performed duplicate.

The epitope that reacts with MAb Cx-99 is carried on an asialyted glycoprotein with a relative molecular mass of 18–37 kD and a pI of 5.3. It apparently does not crossreact with CEA and TA-4 (SCC) Antigen. Also, its physiochemical and antigenic properties are distinct from those of other reported epithelial cell-associated antigens. Tissue polypeptide antigen (TPA) is a 45 kD complex related to cytokeratins number 8, 18 and 19 that is also located in the basal cells of the normal squamous epithelium; however it has rarely been found in keratinised cervical SCC and is absence in the normal endocervical columnar cells (Stegner *et al.*, 1986). MAb 17.13 reacts with a basal cell antigen, but is not present in adenocarcinomas (Randen *et al.*, 1987). MAb 17-1A is a 37 kD glycoprotein, but unlike MAb Cx-99, the epitope that it recognises contains sialic acid residues. Also, the SDS-PAGE banding pattern of the molecule reactive with MAb 17-1A was changed under reducing condition (Gottlinger *et al.*, 1986); therefore, its epitope is structurally different from that recognised by MAb Cx-99 which was not changed under reducing conditions in SDS-PAGE. MAb MOv18 and 19 have a relative molecular weight similar to the antigen that reacts with MAb Cx-99, but they are different in immunohistochemistry (Miotti *et al.*, 1987). The other antigens of SCC include human milk fat globulin (HMFG 1 and 2), epithelial membrane antigen (EMA), VM-2, MAbs CE 400-413, cytokeratins of low molecular weight such as CAM 5.2, and antigens reactive with antibodies SQM1 and Ca 1. However, all of these antigens are different from the antigen recognised by MAb Cx-99 in molecular weight, biochemical characteristics and tissue distribution (Fray *et al.*, 1984; Bamford *et al.*, 1983; Mortenn *et al.*, 1985; Koprowska *et al.*, 1986; Bobrow *et al.*, 1986; Boeheim *et al.*, 1985; Ashall *et al.*, 1982).

In summary, we have established a MAb that recognises an antigen present only in epithelial cells. Based on its pattern of reactivity, we suggest that MAb Cx-99 may be useful for the rapid diagnosis of cervical carcinomas and the detection of micrometastases or microinvasion. Since it is shed by cell lines in culture, it may be present in the sera of patients with SCC or with adenocarcinomas.

**Figure 4** Detection of the antigen reactive with MAb Cx-99 in the culture medium of various cell lines. **a**, A competitive inhibition ELISA test, 1 × and 10 × dilutions of the culture medium were used. A: SIHA culture medium was replaced by PBS (positive control). B: MAb Cx-99 was replaced by PBS (negative control). **b**, ELISA assay of SIHA supernatant generated in serum-free medium. This antigen preparation was used to coat the wells, and is reacted with MAb Cx-99 (□). Replacing the antibody with PBS eliminated the reactivity with SIHA supernatant-coated wells (absorbance <0.09). The titration curve of MAb Cx-99 with SIHA cells is also shown (*).

We acknowledge the valuable advices of Dr Chun Lee of the Northwestern University Medical School, Chicago. The work was supported by grants from the National Science Council of Taiwan, ROC.

### References

ASHALL, F., BRAMWELL, M.E. & HARRIS, H. (1982). A new marker for human cancer cells. 1. The Ca antigen and the Ca1 antibody. *Lancet*, **ii**, 1.

BAMFORD, P.N., ORMEROD, M.G, SLOANE, J.P. & WARBURTON, M.J. (1983). An immunohistochemical study of the distribution of epithelial antigens in the uterine cervix. *Obstet. Gynecol.*, **61**, 603.

BOBROW, L.G., MAKIN, C.A., LAW, S. & BODMER, W.F. (1986). Expression of low molecular weight cytokeratin proteins in cervical neoplasia. *J. Pathol.*, **148**, 135.

BOEHEIM, K., SPEAK, J.A., FREII, III E. & BERNAL, S.D. (1985). SQM1 antibody defines a surface membrane antigen in squamous carcinoma of the head and neck. *Int. J. Cancer*, **36**, 137.

CHANG, C.M., LIN, Y.M., LEE, T.W. & 6 others (1983). Induction of plasma protein secretion in a newly established human hepatoma cell line. *Mol. Cell. Biol.*, **3**, 1133.

CHEN, K.Y. (1986). Current status of cancer epidemiology in Taiwan. In *Clinical Oncology (Annual Report)*, Chen, K.Y. (ed.), p. 1. Cancer Treatment Center, Veterans General Hospital: Taipei.

DALLENBACH-HELLWEG, G. & POULSON, H. (1990). Atlas of histopathology of the cervix uteri. In *Atlas of Histopathology of the Cervix Uteri*, Dallenbach-Hellweg, C.G. & Poulsen, H. (eds), p. 14. Springer-Verlag: Berlin.

ENGLISH, D. & ANDERSEN, B.R. (1974). Single-step separation of red blood cells, granulocytes and mononuclear leukocytes on discontinuous density gradient of Ficoll-Hyaque. *J. Immunol. Methods*, **5**, 249.

FRAY, R.E., HUSAIN, O.A.N., TO, A.C.W. & 4 others (1984). The value of immunohistochemical markers in the diagnosis of cervical neoplasia. *Br. J. Obstet. Gynecol.*, **91**, 1037.

GOTTLINGER, H.G., FUNKE, H., JOHNSON, J.P., GOKEL, M. & RIETHMULLER, G. (1986). The epithelial cell surface antigen 17-1A, a target for antibody-mediated tumor therapy: its biochemical nature tissue distribution and recognition by different monoclonal antibodies. *Int. J. Cancer*, **38**, 47.

HO, C.K., CHIANG, H., LI, S.Y., YUAN, C.C. & NG, H.T. (1987). Establishment and characterization of a tumorigenic trophoblast-like cell line from a human placenta. *Cancer Res.*, **47**, 3220.

HSU, S.M. & RAINE, L. (1981). The use of avidin-biotin-peroxidase complex (ABC) in immunoperoxidase technique: a comparison between ABC and unlabeled antibody (PAP) procedures. *J. Histochem. Cytochem.*, **29**, 577.

HU, C.P., HAN, S.H., LUI, W.Y. & 8 others (1986). Monoclonal antibodies against antigen expressed on human hepatocellular carcinoma cells. *Hepatology*, **6**, 185.

JOHNSTONE, A. & THORPE, R. (1987). Immunocytochemistry and immunohistochemistry. In *Immunochemistry in Practice*, Johnstone, A. & Thorpe, R. (eds), p. 261. Blackwell: Oxford.

KATO, H., MIYAUCHI, F., MORIOKA, H., FUJINO, T. & TORIGOE, T. (1979). Tumor antigen of human cervical squamous cell carcinoma, correlation of circulating levels with disease progress. *Cancer*, **43**, 585.

KO, J.L., CHANG, C.M., CHAO, K.C., WU, K.D., NG, H.T & HU, C.P. (1980). Establishment and characterization of human cervical carcinoma cells in continuous culture. *China J. Microbiol. Immunol.*, **13**, 273.

KOPROWSKA, I., ZIPFEL, S., ROSS, A.H. & HERLYN, M. (1986). Development of monoclonal antibodies that recognize antigens associated with human cervical carcinoma. *Acta Cytol.*, **30**, 207.

LAEMMLI, U.K. (1970). Cleavage of structural proteins during the assembly of the head of bacteriophage T4. *Nature*, **227**, 680.

LOWRY, O.H., ROSEBROUGH, N.J., FARR, A.L. & RANDALL, R.J. (1951). Protein measurement with the folin phenol reagent. *J. Biol. Chem.*, **193**, 265.

MIOTTI, S., CANEVARI, S., MENARD, S. & 4 others (1987). Characterization of human ovarian carcinoma-associated antigens defined by novel monoclonal antibodies with tumor-restricted specificity. *Int. J. Cancer*, **39**, 297.

MORTENN, V.B., SCHREIBER, A.B., SORIERO, O., MCMILLAN, W. & ALLISONI, A.C. (1985). A monoclonal antibody against basal cells of human epidermis potential use in the diagnosis of cervical neoplasia. *J. Clin. Invest.*, **76**, 1978.

RANDEN, R., WHITE, C.F., GOTTFRIED, T.G. & 4 others (1987). Reactivity of monoclonal antibody 17.13 with human squamous cell carcinoma and its application to tumor diagnosis. *Cancer Res.*, **47**, 5684.

RUTANEN, E.M., LINDGREN, J., SIPPONEN, P., STENMAN, U.H., SAKSELA, E. & SEPPALA, M. (1978). Carcinoembryonic antigen in malignant and nonmalignant gynecologic tumors. *Cancer*, **42**, 581.

STEGNER, H.E., KUHLER, C. & LONING, T. (1986). Tissue polypeptide antigen and keratins in cervical neoplasia. *Int. J. Gynecol. Pathol.*, **5**, 23.

TOWBIN, H., STAEHELIN, T. & GORDON, J. (1979). Electrophorectic transfer of proteins from polyacrylamide gels to nitrocellulose sheets: procedure and some applications. *Proc. Natl Acad. Sci.*, **76**, 4350.

TSAI, L.C., CHEN, H.M., YEH, S.H., YUAN, C.C., TSAO, D. & HAN, S.H. (1988). Localization of human colorectal carcinoma xenografts in mice using radiolabeled monoclonal antibody to carcinoembryonic antigen. *Diag. Clin. Immunol.*, **5**, 332.

TSAI, L.C., WANG, F.M., PAN, L.C., PERNG, A.C. & HAN, S.H. (1985). Immunological characteristics of monoclonal antibodies against human carcinoembryonic antigen (CEA). *Proc. Natl Sci. Counc. B. ROC.*, **9**, 287.